# Purification, Characterization, and Gene Expression of Rice Endo-β-*N*-Acetylglucosaminidase, Endo-Os

**DOI:** 10.3389/fpls.2021.647684

**Published:** 2021-08-10

**Authors:** Megumi Maeda, Naoko Okamoto, Norie Araki, Yoshinobu Kimura

**Affiliations:** ^1^Department of Biofunctional Chemistry, Graduate School of Environmental and Life Science, Okayama University, Okayama, Japan; ^2^Department of Tumor Genetics and Biology, Graduate School of Medical Sciences, Kumamoto University, Kumamoto, Japan

**Keywords:** endo-β-*N*-acetylglucosaminidase, free *N*-glycans, *Oryza sativa*, ER associated degradation, peptide:*N*-glycanase

## Abstract

In the endoplasmic reticulum-associated degradation system of plant and animal cells, high-mannose type free *N*-glycans (HMT-FNGs) are produced from misfolded glycoproteins prior to proteasomal degradation, and two enzymes, cytosolic peptide:*N*-glycanase (cPNGase) and endo-β-*N-*acetylglucosaminidase (endo-β-GlcNAc-ase), are involved in the deglycosylation. Although the physiological functions of these FNGs in plant growth and development remain to be elucidated, detailed characterization of cPNGase and endo-β-GlcNAc-ase is required. In our previous work, we described the purification, characterization, and subcellular distribution of some plant endo-β-GlcNAc-ases and preliminarily reported the gene information of rice endo-β-GlcNAc-ase (Endo-Os). Furthermore, we analyzed the changes in gene expression of endo-β-GlcNAc-ase during tomato fruit maturation and constructed a mutant line of *Arabidopsis thaliana*, in which the two endo-β-GlcNAc-ase genes were knocked-out based on the Endo-Os gene. In this report, we describe the purification, characterization, amino acid sequence, and gene cloning of Endo-Os in detail. Purified Endo-Os, with an optimal pH of 6.5, showed high activity for high-mannose type *N*-glycans bearing the Manα1-2Manα1-3Manβ1 unit; this substrate specificity was almost the same as that of other plant endo-β-GlcNAc-ases, suggesting that Endo-Os plays a critical role in the production of HTM-FNGs in the cytosol. Electrospray ionization-mass spectrometry analysis of the tryptic peptides revealed 17 internal amino acid sequences, including the C terminus; the N-terminal sequence could not be identified due to chemical modification. These internal amino acid sequences were consistent with the amino acid sequence (UniProt ID: Q5W6R1) deduced from the *Oryza sativa* cDNA clone AK112067 (gene ID: *Os05g0346500*). Recombinant Endo-Os expressed in *Escherichia coli* using cDNA showed the same enzymatic properties as those of native Endo-Os.

## Introduction

Free *N*-glycans (FNGs) occur ubiquitously in developing or proliferating plants, such as seedlings, developing seeds or fruits, and cells in culture. Plant FNGs are classified into two subclasses based on the reducing end-side structures: the GN1 type FNGs (with one GlcNAc residue at their reducing end-side) and the GN2 type FNGs (with the di-*N*-acetylchitobiosyl unit; Cacan and Verbelt, 1997; Suzuki and Funakoshi, 2006; Maeda and Kimura, 2014). Among the plant FNGs, the GN1 high-mannose type free *N-*glycans (GN1-HMT-FNGs) are believed to be produced from misfolded glycoproteins by the sequential action of two enzymes, cytosolic peptide:*N*-glycanase (cPNGase) and endo-β-N-acetylglucosaminidase (endo-β-GlcNAc-ase; Diepold et al., 2007; Masahara-Negishi et al., 2012). Previous studies (Fischl et al., 2011; Kimura et al., 2011) reported the construction of mutant lines of *Arabidopsis thaliana*, in which two endo-β-GlcNAc-ase genes were knocked-out and the endo-β-GlcNAc-ase activity was completely lost. In the mutant lines, GN1-HMT-FNGs completely disappeared and all the HMT-FNGs produced were GN2-type FNGs, suggesting that endo-β-GlcNAc-ase acts on the products (GN2-HMT-FNGs) of cPNGase but not on the misfolded glycoproteins directly.

To date, many plant endo-β-GlcNAc-ases have been purified and characterized (Yet and Wold, 1988; Nishiyama et al., 1991; Berger et al., 1995; Kimura et al.,1998a,b, 2002). Plant endo-β-GlcNAc-ases showed strong activity against high-mannose type *N*-glycans with the Manα1-2Manα1-3Manβ1 unit but not the plant complex type *N*-glycans with α1-3Fuc and β1-2Xyl residues, indicating that plant endo-β-GlcNAc-ase must have a common subsite for the structural unit Manα1-2Manα1-3Manβ1-4GlcNAcβ1-4GlcNAc (Kimura et al.,1998a,b, 2002). However, the primary structure of plant endo-β-GlcNAc-ase has not been determined because complete purification of the enzyme has not been achieved. In 2002, Suzuki et al. (2002) purified endo-β-GlcNAc-ase from hen oviducts and identified a human ortholog gene based on the purified enzyme. They also reported that there are two orthologs of the animal endo-β-GlcNAc-ase genes in *Arabidopsis thaliana*, suggesting that the genes encoding endo-β-GlcNAc-ase are highly conserved between animals and plants. Kato et al. (2002) identified *Caenorhabditis elegans* endo-β-GlcNAc-ase in the nematode genome database based on the gene information of a fungal endo-β-GlcNAc-ase (Endo-M). They found that the recombinant nematode endo-β-GlcNAc-ase expressed in *Escherichia coli* exhibits almost the same substrate specificity as the Endo-M from *Mucor hiemalis* does (Yamamoto et al., 1994). These eukaryotic endo-β-GlcNAc-ases belong to the GH 85 family, and it is believed that animal and plant endo-β-GlcNAc-ases are involved in the endoplasmic reticulum-associated degradation system and function in the cytosol (Suzuki and Funakoshi, 2006; Maeda and Kimura, 2014).

As described above, we constructed an *A. thaliana* line in which the two endo-β-GlcNAc-ase genes were knocked-out (Kimura et al., 2011), identified a tomato endo-β-GlcNAc-ase gene, and analyzed the changes in gene expression during fruit ripening (Nakamura et al., 2009). For these experiments, we used gene information of the rice endo-β-GlcNAc-ase (Endo-Os), which was found in the rice genome database research based on the information of some internal amino acid sequences of the purified enzyme. Although we reported the amino acid sequence and subcellular localization of Endo-Os previously (Kimura, 2007), we did not report the complete purification, characterization, substrate specificity, and internal amino acid sequences of Endo-Os in detail. Here, we describe the purification and characterization of rice endo-β-GlcNAc-ase and heterologous expression of the Endo-Os gene.

## Materials and Methods

### Materials

The rice k-1 cell line established from *Oryza sativa* L. cv. Nipponbare was kindly gifted by H. Nishimura and the late Professor K. Kasamo (Research Institute for Bioresources, Okayama University). DEAE-cellulose was purchased from Sigma (St. Louis, MO, United States), and Butyl-TOYOPEARL and TSK-Gel G3000SWXL columns (0.78 × 30 cm) were purchased from Tosoh (Tokyo, Japan). A Shodex PH-814 column (0.8 × 7.5 cm) was purchased from Showa Denko (Tokyo, Japan). A Cosmosil 5C18-AR column (0.6 × 25 cm) was purchased from Nacalai Tesque (Kyoto, Japan). Ni-NTA His•Bind^®^ resin and GST•Bind^TM^ resin were purchased from Novagen^®^. Authentic PA-sugar chains were prepared as described in our previous studies (Kimura et al., 1988, 1997, 2000).

### Assay System of Endo-β-GlcNAc-ase Activity

**FIGURE 1:**
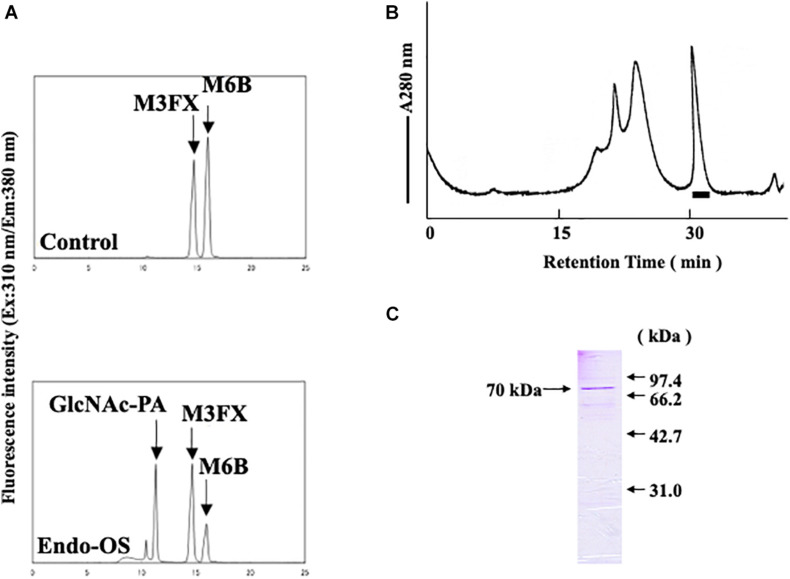
Final purification and SDS-PAGE of the Endo-β-GlcNAc-ase. **(A)** Assay of endo-β-GlcNAc-ase activity on RP-HPLC; **(B)** partially purified endo-β-GlcNAc-ase was applied to the Tsk-Gel G3000SWXL column (7.8 × 600 mm) equilibrated with 25 mM MES at pH 6.7, containing 5 mM of EDTA, 5 mM of DTT, and 0.15 M NaCl, and eluted with the same buffer at a flow rate of 0.5 mL/min. The bar indicates endo-β-GlcNAc-ase activity; and **(C)** proteins were separated using SDS-PAGE on a 15% acrylamide gel under reduced conditions with 5% 2-mercaptoethanol. Proteins on the gel were stained with Coomassie Brilliant Blue R-250.

As shown in Figure 1A, endo-β-GlcNAc-ase activity was assayed using M6B [Manα1-6(Manα1-3)Manα1-6(Manα1-2Manα1-3)Manβ1-4GlcNAcβ1-4GlcNAc-PA] as a substrate and M3FX [Manα1-6(Manα1-3)(Xylβ1-2)Manβ1-4GlcNAcβ1-4(Fucα1-3)GlcNAc-PA] as an internal standard. An enzyme solution (20 μL) was mixed with M6B and M3FX (approximately 100 and 120 pmol, respectively) in 0.5 M MES buffer (25 μL) at pH 6.0, containing 10 mM EDTA. After incubation at 37°C for 2 h, the reaction was stopped by heating at 100°C for 3 min. After centrifugation, an aliquot (30 μL) of the resulting supernatant was analyzed using a Jasco 880-PU HPLC apparatus with a Jasco Intelligent spectrofluorometer and Cosmosil 5C18-AR column. The PA-sugar chains (M6B, M3FX, and GlcNAc-PA) were eluted and separated by increasing the acetonitrile concentration in 0.02% TFA linearly from 0 to 6% for 25 min at a flow rate of 1.2 mL/min. The eluate was monitored using a spectrofluorometer at an excitation wavelength of 310 nm and an emission wavelength of 380 nm. One unit of the enzyme was defined as the amount that hydrolyzes 1 nmol of the substrate per min at 37°C.

### Purification of Endo-*β*-GlcNAc-ase

Purification of the enzyme was performed at 0–4°C. The protein concentration was determined by measuring the light absorption at 280 nm using bovine serum albumin (BSA) as a standard.

#### Preparation of a Crude Enzyme

The rice cultured cells (1,039 g) were disrupted using a Teflon homogenizer (60 rpm and 10 strokes) in 2.1 L of 50 mM Tris–HCl buffer, pH 7.8. The homogenate was filtered through gauze and centrifuged at 8,000 rpm for 20 min. The resulting supernatant was pooled and saturated with ammonium sulfate. The proteins precipitated using 80% saturated ammonium sulfate were dissolved in a small amount of 50 mM Tris–HCl buffer (pH 7.8) and dialyzed against the same buffer. The resulting dialyzate was collected as a crude enzyme.

#### DEAE-Cellulose Column Chromatography

The crude enzyme (270 mL) was applied to a DEAE-cellulose column (4.0 × 80 cm) equilibrated with 50 mM Tris–HCl buffer at pH 7.8. After the column was washed with 50 mM Tris–HCl buffer containing 50 mM NaCl and with the same buffer containing 0.1 M NaCl, the enzyme active fraction was eluted with the same buffer containing 0.2 M NaCl. The fraction with enzyme activity was pooled and saturated with ammonium sulfate.

#### Butyl-TOYOPEARL Column Chromatography

The proteins precipitated using 100% saturated ammonium sulfate were dissolved in a small amount of 50 mM Tris–HCl buffer (pH 7.8) and dialyzed against the same buffer. Ammonium sulfate (2.0 M final concentration) was added to the enzyme solution (30 mL) and directly applied to a Butyl-TOYOPEARL column (3.0 × 35 cm) equilibrated with 50 mM Tris–HCl buffer at pH 7.8 containing 2.0 M ammonium sulfate and eluted with a linear gradient (2.0–0 M) of ammonium sulfate in the same buffer. The fractions with enzyme activity were pooled.

#### Hydrophobic Interaction HPLC

HPLC was performed using a Jasco 880-PU HPLC apparatus with a Jasco Intelligent UV/VIS detector (970 UV) and a Shodex PH-814 column (0.8 × 7.5 cm). The enzyme solution was centrifuged (Amicon Centriprep-30) and 5 mL of the sample was dialyzed against 25 mM MES buffer (pH 6.7) containing 5 mM EDTA and 5 mM DTT. Ammonium sulfate (1.5 M final concentration) was added to the enzyme solution and directly applied to a Shodex PH-814 column equilibrated with 25 mM MES buffer (pH 6.7) containing 1.5 M ammonium sulfate, 5 mM EDTA, and 5 mM DTT and eluted with a linear gradient (1.5–0 M) of ammonium sulfate in the same buffer at a flow rate of 1.0 mL/min.

#### TSK-Gel G3000SWXL Column Chromatography

The enzyme fraction obtained in step 4 was applied to a Shodex TSK-Gel G3000SWXL twin column (0.78. × 60 cm). The enzyme solution was dialyzed against 25 mM MES buffer (pH 6.7) containing 5 mM EDTA, 5 mM DTT, and 0.15 M NaCl. The resulting dialyzate was centrifuged in an Amicon Centriprep-30 up to 780 μL. The concentrated enzyme fraction was applied to the column and eluted with the same buffer at a flow rate of 0.5 mL/min. The enzyme solution was centrifuged in an Amicon Centriprep-30 up to 950 μL.

### Primary Structural Analysis of the Purified Enzyme

The 70-kDa reduced and alkylated protein band on the SDS-PAGE gel was cut and washed with 50 mM ammonium bicarbonate containing 50% acetonitrile and 50 mM ammonium bicarbonate containing 30% acetonitrile successively, and vortexed for 30 min. This treatment was repeated until the colors were completely removed. The gel was dehydrated using 100% acetonitrile on a vortex twice for 15 min. After acetonitrile was evaporated, the gel was digested with trypsin (Promega, United States). Trypsin (0.05 μg) was added to the gel on ice for 30 min and incubated in 50 mM ammonium bicarbonate containing 10% acetonitrile for 16 h at 37°C. The resulting peptides were recovered using 50 mM ammonium bicarbonate containing 30% acetonitrile, 80% acetonitrile, and 100% acetonitrile. The resulting solution was evaporated and concentrated. Trifluoroacetic acid (0.1%) was added to the peptide solution and loaded onto a ZipTip C18 (Millipore, United States) pipette tip. The tip was washed with 0.1% formic acid and the bound peptides were eluted with 50% acetonitrile containing 0.1% formic acid. An Applied Biosystems API QSTAR Pulsar *i*, quadrupole mass spectrometer with an atmospheric pressure ionization ion source was used. It was operated in the positive mode and the ion spray voltage was 950 V. The resulting peptide solution was introduced into the nanospray needle using mechanical infusion at a flow rate of 30 nL/min. The collisionally activated dissociation (CAD) spectrum was measured using argon as the collision gas. The collision energy was 50 eV. Scanning decreased with a step size of 0.1 Da and the CAD daughter ion was recorded from *m/z* 400 to 1,000. The data analysis was performed in Analyst QS and MS/MS Fragment Ion Calculator of Proteomics Toolkit.^1^

### Database Search

A homology search was performed using the Basic Local Alignment Search Tool (BLAST) of the National Center for Biotechnology Information. Additionally, a Rice Full-length cDNA clone search was performed using the Rice Annotation Project Database. A search of protein sequence was performed using the UniProt Knowledgebase.

### Expression of Endo-β-GlcNAc-ase in *E. coli*

A cDNA clone (Clone Name 001-117-D09; Accession No. AK112067) was obtained from the Rice Genome Project of the National Institute of Agrobiological Sciences, Japan, as the developer, and the Rice Genome Resource Center as the provider of the material. The inserted cDNA encoding endo-β-GlcNAc-ase was retrieved with *Xho*I from pME18SFL3-AK112067, and the resulting fragment was subcloned into the two *Xho*I sites of the pET-41b(+) vector (Novagen, Germany). The pET-41b(+)-endo-β-GlcNAc-ase was transformed into *E*. *coli* BL21 (DE3) and the cells were grown in 16 mL of Luria-Bertani medium containing 30 μg/mL kanamycin. After 3 h of shaking culture at 37°C, the cells were collected and grown in 200 mL of Luria-Bertani medium containing 30 μg/mL kanamycin with shaking culture for 24 h at 20°C. The cells were collected and washed with 20 mM Tris–HCl buffer (pH 8.0).

### Purification of Recombinant Endo-β-GlcNAc-ase

Cells (3.6 g) were sonicated in 20 mL of 20 mM Tris–HCl buffer (pH 8.0) containing 0.5 M NaCl and 5 mM imidazole for 10 min at 0°C. The cell extract was centrifuged at 10,000 × *g* for 30 min at 4°C. The resulting supernatant was centrifuged in an Amicon Centriprep-100 up to 10.5 mL. The cell extract was applied to a Ni-NTA His•Bind^TM^ column (1.5 × 3 cm) equilibrated with 20 mM Tris–HCl buffer, pH 8.0, containing 0.5 M NaCl and 5 mM imidazole. After the column was washed with 20 mM Tris–HCl buffer at pH 8.0, containing 0.5 M NaCl and 20 mM imidazole until no further protein was eluted, the recombinant endo-β-GlcNAc-ase was eluted with 20 mM Tris–HCl buffer at pH 8.0, containing 0.5 M NaCl and 200 mM imidazole. The endo-β-GlcNAc-ase active fractions were pooled and added to 5 mM of EDTA and 1 mM of DTT. The enzyme solution was applied to a GST•Bind^TM^ column (1.5 × 1.2 cm) equilibrated with 50 mM Tris–HCl buffer at pH 8.0, containing 0.1 M NaCl, 5 mM of EDTA, and 1 mM of DTT. After washing the column with the same buffer, the recombinant endo-β-GlcNAc-ase was eluted with the same buffer containing 10 mM of reduced glutathione.

### SDS-PAGE

SDS-PAGE was performed using the method of Laemmli under reducing conditions using 5% 2-mercaptoethanol (Laemmli and Favre, 1973). Proteins were stained using Coomassie Brilliant Blue R-250. As standard proteins for the mass calibration, myosin (212 kDa), α_*2*_-macroglobulin (170 kDa), β-galactosidase (116 kDa), transferring (76 kDa), glutamic dehydrogenase (53 kDa), phosphorylase B (97.4 kDa), BSA (66.2 kDa), ovalbumin (42.7 kDa), and carbonic anhydrase (31.0 kDa) were used (Pharmacia, United States).

### Effects of pH and Temperature on Enzyme Activity

The optimum pH of the enzyme was determined after incubation with M6B as a substrate and M3FX as an internal standard in buffers with various pH (4.0–8.5) at 37°C for 30 min. The buffers used were 0.1 M MES (pH 4.0–6.5) and 0.1 M HEPES (pH 7.0–8.5). The optimum temperature of the enzyme was measured with M6B after incubation in 0.5 M MES buffer, pH 6.0, containing 10 mM of EDTA at various temperatures for 30 min.

### Effects of Metal Ions, Detergent, and Chaotropic Ions on Enzyme Activity

The effects of metal ions, detergent, and chaotropic ions on the enzyme were examined after incubation with M6B as a substrate and M3FX as an internal standard in 0.5 M MES buffer, pH 6.0, containing metal ions, detergent, or chaotropic ions at 37°C for 30 min.

### Substrate Specificity

Substrate specificity of the enzyme was examined after incubation with various substrates and GlcNAcβ1-4GlcNAcβ1-4GlcNAcβ1-4GlcNAcβ1-4GlcNAc-PA (GN5) as an internal standard in 0.5 M MES buffer at pH 6.0, containing 10 mM EDTA at 37°C for 30 min.

## Results

### Purification of Endo-β-GlcNAc-ases From Rice Cultured Cells

An endo-β-GlcNAc-ase was purified from rice-cultured cells by a combination of ion-exchange chromatography, hydrophobic interaction chromatography, and gel filtration. The elution profile of the final gel filtration using a TSK-Gel G3000SWXL column and SDS-PAGE analysis of the purified rice endo-β-GlcNAc-ase are shown in Figures 1B,C. A summary of the purification procedure is shown in Table 1. The specific activity of the purified enzyme represented a 1200-fold increase over the crude extract. The molecular mass of the purified endo-β-GlcNAc-ase (nEndo-Os) was estimated to be 70 kDa using SDS-PAGE analysis.

**TABLE 1:** Purification of Endo-β-GlcNAc-ase from rice culture cells.

Steps	Total Optical Density (A280 nm × Volume)	Total activity (units)	Recovery (%)	Specific act. (units / mg)	Purification fold
Crude Extract	24840.00	305.10	100	0.012	1.0
DEAE-Cellulose	588.00	42.00	13.8	0.071	5.9
Butyl-Toyopearl	13.31	28.86	9.5	2.168	180.7
PH-814	2.92	10.03	3.3	3.436	218.2
Tsk-Gel G3000SWXL	0.18	2.76	0.9	15.165	1263.8

*One unit of enzyme activity was defined as the amount of the enzyme releasing 1 nmol of substrate per min at 37°C.*

### Internal Amino Acid Sequences of Purified nEndo-Os

**FIGURE 2:**
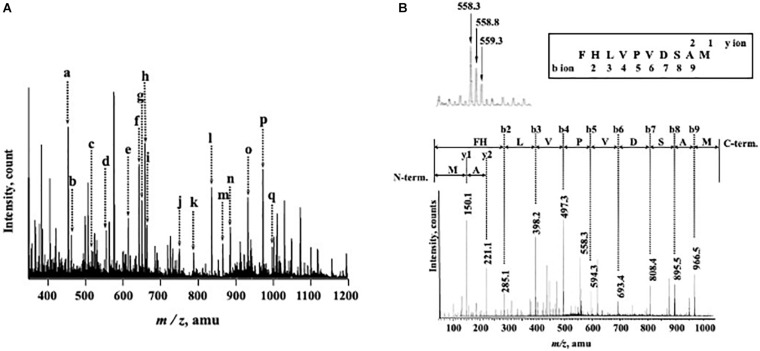
ESI-TOF-MS analysis of tryptic peptides from purified Endo-β-GlcNAc-ase and MS/MS spectrum of peak-d (*m/z* 558.3; C-terminal Peptide). **(A)** Doubly and triply charged tryptic peptides were obtained and further analyzed using MS/MS spectrometry. a, *m/z* 457.5 [(M + 2H)^2+^]; b, *m/z* 466.2 [(M + 2H)^2+^]; c, *m/z* 519.8 [(M + 2H)^2+^]; d, *m/z* 558.3 [(M + 2H)^2+^]; e, *m/z* 617.0 [(M + 3H)^3+^]; f, *m/z* 645.8 [(M + 2H)^2+^]; g, *m/z* 653.3 [(M + 2H)^2+^]; h, *m/z* 661.8 [(M + 2H)^2+^]; i, *m/z* 666.3 [(M + 3H)^3+^]; j, *m/z* 752.9 [(M + 2H)^2+^]; k, *m/z* 789.9 [(M + 2H)^2+^]; l, *m/z* 838.7 [(M + 3H)^3+^]; m, *m/z* 868.4 [(M + 2H)^2+^]; n, *m/z* 887.4 [(M + 3H)^3+^]; o, *m/z* 934.9 [(M + 2H)^2+^]; p, *m/z* 974.5 [(M + 2H)^2+^]; and q, *m/z* 999.0 [(M + 2H)^2+^]. **(B)** MS/MS Spectrum of Peak-c (*m/z* 558.3; C-terminal Peptide).

**FIGURE 3:**
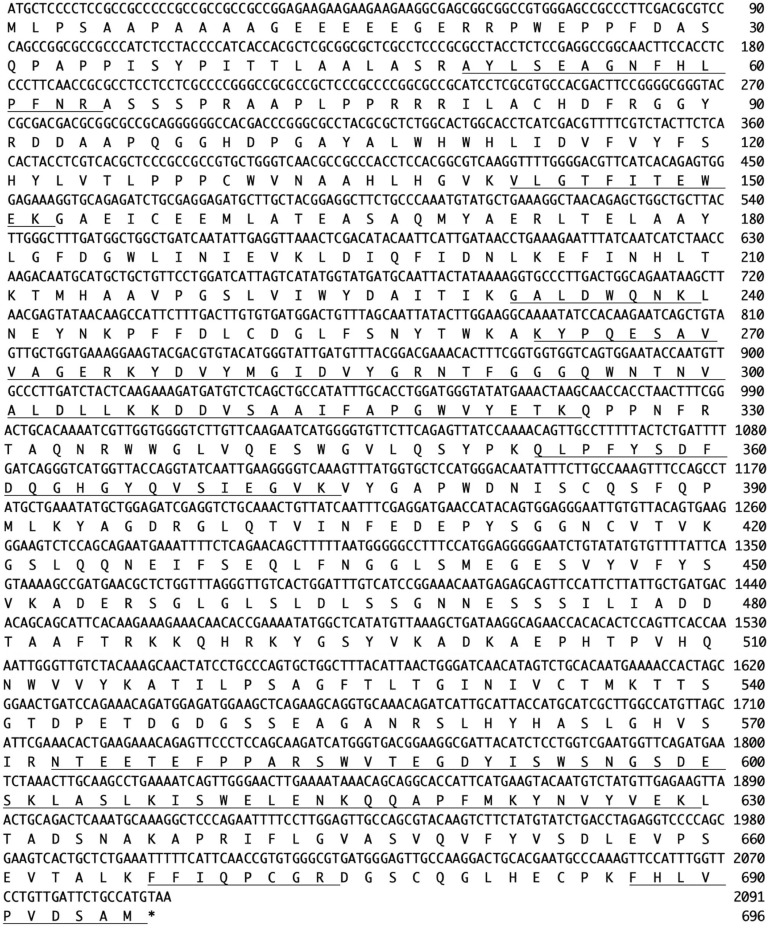
Nucleotide sequence (*Os05g0346500*) and amino acid sequence (UniProt ID: Q5W6R1) of Endo-Os. Underlining indicates identified peptide sequences.

N-Terminal amino acid sequences could not be identified, suggesting that the N-terminus of this enzyme might be modified. Therefore, we attempted to determine the internal amino acid sequences using tryptic peptides of purified nEndo-Os and ESI-MS spectrometry. The divalent and trivalent ions from tryptic peptides were obtained using ESI-TOF-MS analysis (Figure 2A) and were further analyzed using MS/MS spectrometry. In Figure 2B, the result of C-terminal sequence is shown as a typical example of MS/MS analysis. The results of MS/MS analysis of these peptides are summarized in Table 2. As shown in Figure 3, the result of the homology search using BLAST indicated that all these sequences coincide with the deduced amino acid sequences (UniProt ID: Q5W6R1) in a gene (*Os05g0346500*) product, of which putative function is believed to be a glycoside hydrolase family 85 domain-containing protein.

**TABLE 2:** MS/MS analysis of internal amino acid sequence of purified endoglycosidase.

Mass (*m / z*)	Peptide Sequence
a, 457.5 [(M+2H)2+]	YNVYVEK
b, 466.2 [(M+2H)2+]	GALDWQNK
c, 519.8 [(M+2H)2+]	FFIQPXGR
d, 558.3 [(M+2H)2+]	FHLVPVDSAM
e, 617.0 [(M+3H)3+]	ISWELENKQQAPFMK
f, 645.8 [(M+2H)2+]	NTEETEFPPAR
g, 653.3 [(M+2H)2+]	YPQESAVVAGER
h, 661.8 [(M+2H)2+]	VLGTFITEWEK
i, 666.3 [(M+3H)3+]	KDDVSAAIFAPGWVYETK
j, 752.9 [(M+2H)2+]	AKYPQESAVVAGER
k, 789.9 [(M+2H)2+]	KYDVYMGIDVYGR
1, 838.7 [(M+3H)3+]	QLPFYSDFDQGHGYQVSIEXXK
m, 868.4 [(M+2H)2+]	AYLSEAGNFHLPFNR
n, 887.4 [(M+3H)3+]	SWVTEGXXXXXXXXXXXSKLASLK
o, 934.9 [(M+2H)2+]	DDVSAAIFAPXWVYETK
p, 974.5 [(M+2H)2+]	NTFGGGQWNTNVALDLLK
q, 999.0 F(M+2H)2+]	KDDVSAAIFAXXXVYETK

*X means not identified.*

### Expression of Recombinant Endo-Os in *E. coli* and Purification

**FIGURE 4:**
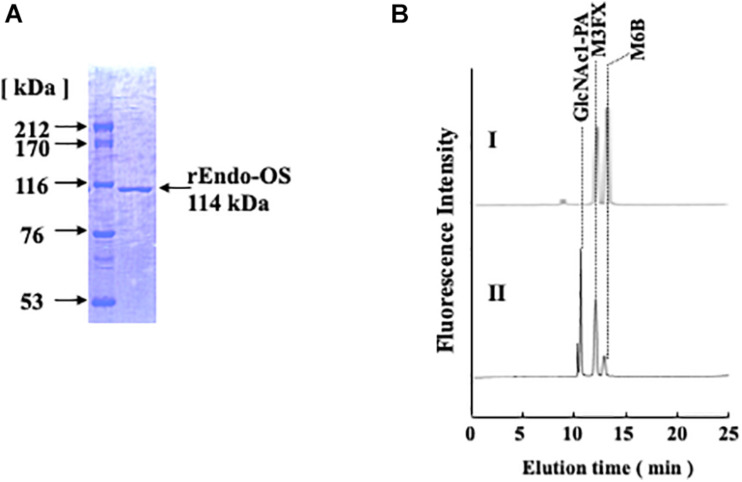
Purification of recombinant Endo-β-GlcNAc-ase. **(A)** Proteins were separated using SDS-PAGE on a 7.5% acrylamide gel under reduced conditions using 1 mM DTT. Proteins on the gel were stained with Coomassie Brilliant Blue R-250; **(B)** Assay of endo-β-GlcNAc-ase activity on RP-HPLC. I, Control; II, Enzyme digestion.

To confirm whether the *Os05g0346500* gene codes for nEndo-Os, the putative endo-β-GlcNAc-ase gene was obtained from the cDNA clone AK112067, cloned into the pET-41b(+) vector, and expressed in *E. coli* BL21 (DE3). The expressed recombinant Endo-Os (rEndo-Os), which was tagged with eight histidines (His8), and glutathione S-transferase (GST) in the N-terminal region were purified using a Ni-NTA His•Bind^®^ column followed by a GST•Bind^TM^ column. The purified recombinant protein with a molecular mass of 114 kDa (Figure 4A) exhibited endo-β-GlcNAc-ase activity (Figure 4B), indicating that the putative function of the *Os05g0346500* gene (the cDNA clone AK112067) must encode Endo-Os, and that the gene is expressed and involved in the release of high-mannose type *N*-glycans in rice cells.

### Effects of pH and Temperature on Enzyme Activity

**FIGURE 5:**
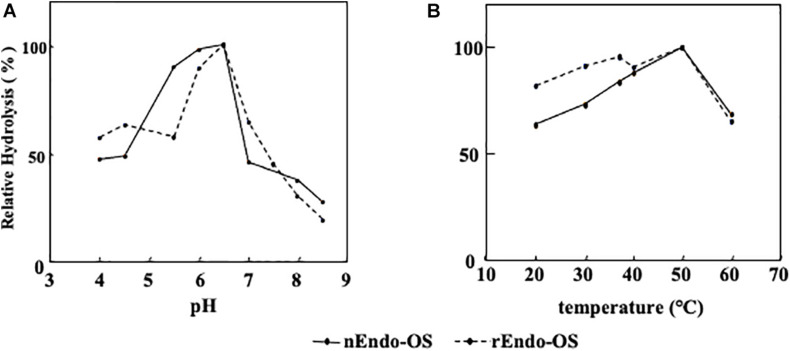
Effects of pH and temperature on Endo-Os activity. Optimum pH and temperature were determined using M6B as a substrate and M3FX as an internal standard. **(A)** Optimum pH was determined after incubation in buffers with various pH (0.1 M MES buffer for pH 4.0–6.5 and 0.1 M HEPES buffer for pH 7.0–8.5) at 37°C for 30 min. **(B)** Optimum temperature was determined after incubation in 0.5 M MES buffer at pH 6.0, containing 10 mM of EDTA at various temperatures for 30 min.

The effects of pH and temperature on the activity of nEndo-Os and rEndo-Os were determined using M6B (150 pmol) as a substrate and M3FX (150 pmol) as an internal standard. As shown in Figure 5, both enzymes had an optimum pH of 6.5 and an optimum temperature of approximately 50°C.

### Effects of Various Additives

**FIGURE 6:**
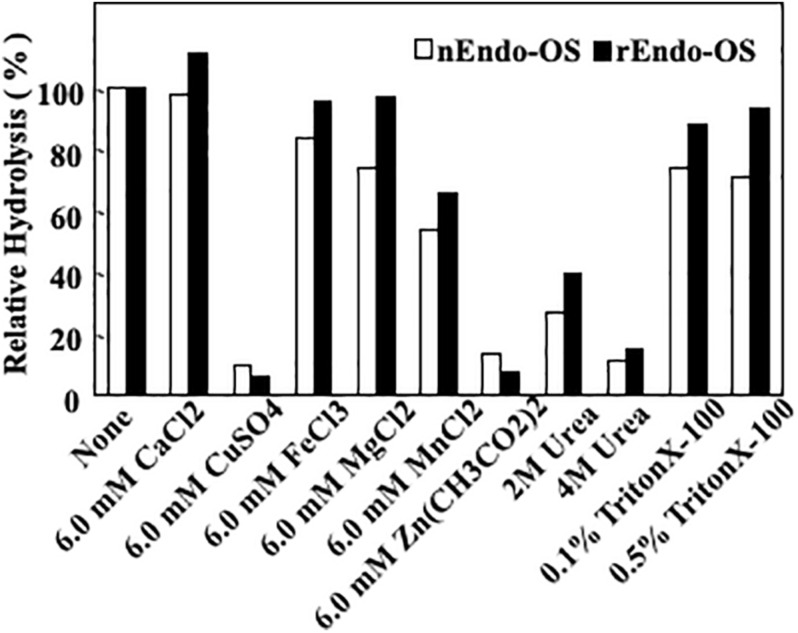
Effects of metal ions, detergent, and chaotropic ions on Endo-Os activity. Enzymes were incubated in 0.5 M MES buffer at pH 6.0, containing metal ions (6 mM), detergent (2 and 4 M), or chaotropic ions (0.1 and 0.5%) at 37°C for 30 min. Enzyme activity was determined using M6B as a substrate and M3FX as an internal standard.

The effects of various additives, such as metal ions, detergents, and chaotropic ions, on enzyme activity were examined, as shown in Figure 6. The enzymes (nEndo-Os and rEndo-Os) were incubated with various additives in 0.5 M MES buffer at pH 6.0, at 37°C for 30 min, and the residual activity was assayed. Both enzymes exhibited similar properties. Cu^2+^ and Zn^2+^ (6.0 mM) inhibited the activity by 90%, while Mn^2+^ was inhibited it by 46%. Since EDTA (10 mM) had no effect on the enzyme activities (data not shown), endo-β-GlcNAc-ase should require no metal ions for activity. A chaotropic reagent, urea, significantly inhibited both endo-β-GlcNAc-ase activities (approximately 70% inhibition by 2 M and approximately 80% inhibition by 4 M urea), while Endo-Os retained almost complete activity in both 0.1 and 0.5% Triton X-100.

### Substrate Specificity of nEndo-Os and rEndo-Os

The substrate specificities of the rice endo-β-GlcNAc-ase enzymes (native Endo-Os and recombinant Endo-Os) were examined using various PA-sugar chains (both high-mannose type and plant complex type *N*-glycans) as substrates. A summary of the substrate specificity analysis is shown in Table 3. nEndo-Os and rEndo-Os showed strong activity against the high-mannose type free *N-*glycans (HMT-FNGs) with the Manα1-2Manα1-3Manβ1-unit, such as M5B, M6B, M7A, M7B, M7D, M8A, M8C, and M9A, and exhibited moderate activity toward GNM5, M4C, and M5A, but neither enzyme could hydrolyze the *N*-acetylchitobiosyl linkage in the plant complex type *N-*glycans. The rates of hydrolysis for these high-mannose type *N*-glycans were comparable to each other, as shown in Table 3. The significant difference in the hydrolysis rate between M5A and M5B suggested that the plant endo-β-GlcNAc-ase must have a specific subsite for the Manα1-2Manα1-3Manβ1-4GlcNAcβ1-4GlcNAc unit to enhance the hydrolytic reaction. Furthermore, in addition to the pentasaccharide unit, the linkage position of the α1-2 mannose residue at the non-reducing end gave different effects on the endo-β-GlcNAc-ase activity; α1-2 mannose residue linked to the α1-6Man in M7A and to α1-2Man in M7B provided the positive effect but the α1-2 mannose residue linked to α1-3Man in M7D provided a negative effect. In contrast, the core pentasaccharide of *N-*glycan [Manα1-6(Manα1-3)Manβ1-4GlcNAcβ1-4GlcNAc] and trisaccharide with only one mannosyl residue (Manβ1-4GlcNAcβ1-4GlcNAc) were hardly hydrolyzed.

**TABLE 3:** Comparison of substrate specificities of Endo-OS.

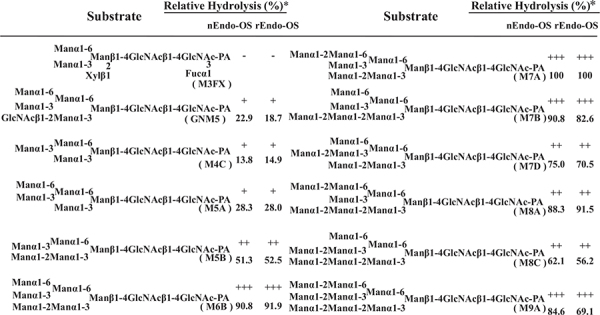

*Substrate : 100 pmol, 37°C, 30 min, 0.5 M MES buffer, pH 6.0, with 10 mM EDTA*

*−: not hydrolyzed, + : hydrolyzed (10–35%), + ⁣ + : hydrolyzed (36–65%), + ⁣ + ⁣ + : hydrolyzed (66–100%)*

*^∗^:The value obtained with M7A was taken as 100.*

**FIGURE 7:**
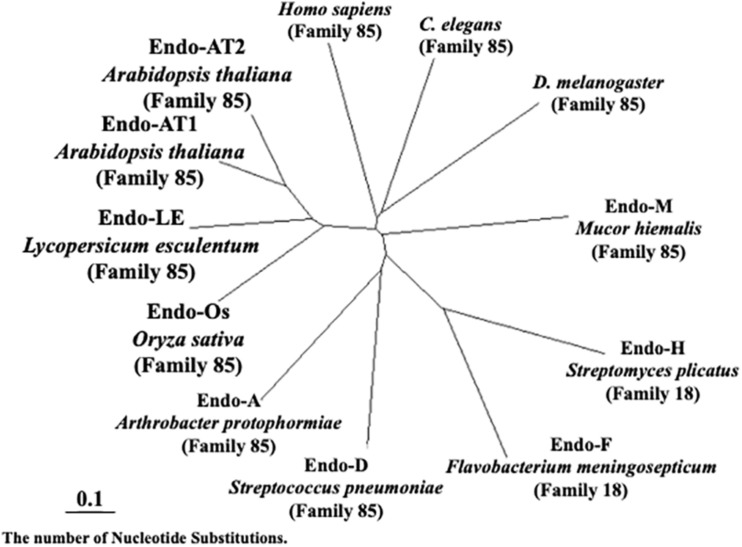
Neighbor-joining phylogenetic tree of amino acid sequences of Endo-β-GlcNAc-ase. Amino acid sequences of *Oryza sativa* (Endo-Os, Q5W6R1) used the putative endo-β-GlcNAc-ase sequence. *Arabidopsis thaliana-1* (Endo-AT1, F4JZC2), *A. thaliana-2* (Endo-AT2, Q9SRL4), *Lycopersicum esculentum* (Endo-LE, A0A3Q7HNF7), *Homo sapiens* (Q8NFI3), *C. elegans* (Q8TA65), *D. melanogaster* (Q9VX51), *Mucor hiemalis* (Endo-M, Q9C1S6), *Streptomyces plicatus* (Endo-H, Q93HW0), *Flavobacterium meningosepticum* (Endo-F, P36911), *Streptococcus pneumoniae* (Endo-D, Q93HW0), and *Arthrobacter protophormiae* (Endo-A, Q9ZB22).

**FIGURE 8:**
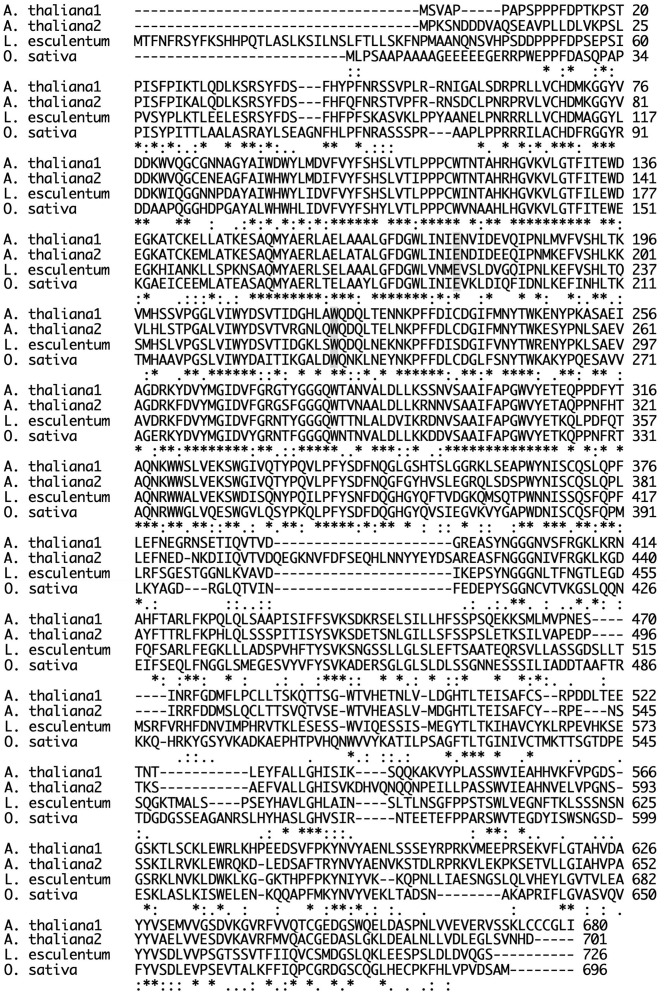
Comparison of sequences of plant Endo-β-GlcNAc-ase. Endo-AT1 and -AT2, *Arabidopsis thaliana* endo-β-GlcNAc-ase (F4JZC2 and Q9SRL4); Endo-LE, *Lycopersicum esculentum* endo-β-GlcNAc-ase (A0A3Q7HNF7); Endo-Os, *Oryza sativa* putative endo-β-GlcNAc-ase (Endo-Os, Q5W6R1). A gray box indicates an important site; E, endo-β-GlcNAc-ase; W, transglycosylation.

As shown in Figure 7, plant endo-β-GlcNAc-ases form a cluster that is slightly distant from animal endo-β-GlcNAc-ases, suggesting that the plant enzyme and the animal enzymes may carry certain different physiological function in addition to the hydrolytic activity of HMT-GN2-FNGs. The deduced amino acid sequence of Endo-Os showed high homology with three endo-β-GlcNAc-ases in *A. thaliana* (Endo-AT1 and Endo-AT2) and *L. esculentum* (Endo-LE; Figure 8). A phylogenetic tree based on the full amino acid sequences of various endo-β-GlcNAc-ases was constructed using the neighbor-joining method (Saitou and Nei, 1987).

## Discussion

In this study, we purified a rice Endo-Os and determined the internal amino acid sequences of purified Endo-Os by ESI-MS analysis. To identify the endo-β-GlcNAc-ase gene, *O. sativa* Endo-Os was completely purified from the cultured rice cells. Since the N-terminal amino acid sequence of the purified enzyme could not be determined due to some chemical modification, we analyzed the internal amino acid sequences using some tryptic peptides by ESI-TOF-MS. All internal sequences determined from the purified Endo-Os were found in the deduced amino acid sequence of the *O. sativa* cDNA clone AK112067 (gene ID: *Os05g0346500*), suggesting that the gene encodes Endo-Os (UniProt ID: Q5W6R1). The recombinant Endo-Os tagged with eight histidine residues and GST, which was expressed in *E. coli*, showed endo-β-GlcNAc-ase activity, indicating that the gene (*Os05g0346500*) encodes the rice endo-β-GlcNAc-ase. Several plant endo-β-GlcNAc-ases have been purified (or partially purified), and their enzymatic properties, including substrate specificity, have been reported in previous studies (Kimura et al.,1998a,b, 2002); however, the primary structure or internal amino acid sequences have not been analyzed and the heterologous expression of rice endo-β-GlcNAc-ase has not been achieved. To our knowledge, this study is the first to report the identification of a plant endo-β-GlcNAc-ase gene based on the information of internal amino acid sequences determined from the purified enzyme. The native Endo-Os and the recombinant Endo-Os showed almost the same substrate specificity and high activity toward the high-mannose type *N*-glycans bearing the Manα1-2Manα1-3Manβ1 unit. It is noteworthy that this type of *N*-glycan is often linked to glycoproteins that are misfolded in the ER and transported into the cytosol.

The double knock-out mutants of Endo-AT1 (*At5g05460*) and Endo-AT2 (*At3g11040*) genes in *A. thaliana* showed no significant changes in phenotype under normal growing conditions. However, most HMT-FNGs found in the mutant lines were of the GN2 type, and the GN1 type, which is predominant in the wild-type plant, was lost, suggesting that the major substrates for plant endo-β-GlcNAc-ase are HMT-GN2-FNGs and not misfolded glycoproteins with high-mannose type *N*-glycans (Fischl et al., 2011; Kimura et al., 2011). It is believed that in animal cells, HMT-GN1-FNG produced by endo-β-GlcNAc-ase in the cytosol is hydrolyzed by cytosolic α-mannosidase, which is absent in plants, and is then transported to lysosomes for further degradation (Suzuki and Funakoshi, 2006). Since plants lack such cytosolic α-mannosidases, the degradation pathway of HMT-GN1-FNGs has remained obscure. However, it has recently been suggested that HMT-GN1-FNGs are transported back into the ER and then secreted into the extracellular space via glycan processing in the Golgi apparatus (Katsube et al., 2021). Furthermore, we found that HMT-FNGs (both GN1 and GN2 types) are involved in inhibiting β-amyloid fibril formation (Tanaka et al., 2015), suggesting a possibility that these HMT-FNGs transported back into the ER mayplay an important role in facilitating protein folding or inhibiting protein aggregation. On the other hand, it has long been postulated that FNGs function as signaling molecules involved in plant growth or development (Priem and Gross, 1992; Yunovitz and Gross, 1994); however, the putative functions remain to be confirmed.

The physiological significance of plant endo-β-GlcNAc-ase remains to be elucidated, since the suppression of endo-β-GlcNAc-ase activity in plants resulted in no significant changes in phenotype or harmful effects (Fischl et al., 2011; Kimura et al., 2011; Kapusi et al., 2017; Shirai et al., 2019). Therefore, to clarify the physiological importance of plant endo-β-GlcNAc-ase, it may be necessary to generate transgenic plants overexpressing endo-β-GlcNAc-ase.

## Data Availability Statement

The datasets presented in this study can be found in online repositories. The names of the repository/repositories and accession number(s) can be found below: UniProt database under the accession code Q5W6R1 (https://www.uniprot.org/uniprot/Q5W6R1).

## Author Contributions

MM carried out the experiment and wrote the manuscript with support from NO and NA helped carry out the MS and MS/MS analysis. YK supervised the findings of this work. All authors contributed to the article and approved the submitted version.

## Conflict of Interest

The authors declare that the research was conducted in the absence of any commercial or financial relationships that could be construed as a potential conflict of interest.

## Publisher’s Note

All claims expressed in this article are solely those of the authors and do not necessarily represent those of their affiliated organizations, or those of the publisher, the editors and the reviewers. Any product that may be evaluated in this article, or claim that may be made by its manufacturer, is not guaranteed or endorsed by the publisher.

## Footnotes

1
http://db.systemsbiology.net/

## Abbreviations

DTT: dithiothreitol
endo-β-GlcNAc-ase: endo-β-Nacetylglucosaminidase
ESI-TOF-MS: electrospray ionization-time-of flight mass spectrometry
FNG: free N-glycan
Fuc: L-fucose
GlcNAc: N-acetyl-D-glucosamine
GST: glutathione S-transferase
HEPES: 2-[4-(2-hydroxyethyl)-1-piperazinyl] propanesulfonic acid
HMT-FNG: high-mannose type free N-glycan
HRP: horseradish peroxidase
Man: D-mannose
MES: 2-morpholinoethanesulfonic acid
MS/MS: tandem mass
M3FX: Manα1-6(Manα1-3)(Xylβ1-2)Manβ1-4GlcNAcβ1-4(Fuca1-3)GlcNAc-PA
M5A: Manα1-6(Manα1-3)Manα1-6(Manα1-3)Manβ1-4GlcNAcβ1-4GlcNAc-PA
M5B: Manα1-3Manα1-6(Manα1-2Manα1-3)Manβ1-4GlcNAcβ1-4GlcNAc-PA
M6B: Manα1-6(Manα1-3)Manα1-6(Manα1-2Manα1-3)Manβ1-4GlcNAcβ1-4GlcNAc-PA
M7A: Manα1-2Manα1-6(Manα1-3)Manα1-6(Manα1-2Manα1-3)Manβ1-4GlcNAcβ1-4GlcNAc-PA
M7B: Manα1-6(Manα1-3)Manα1-6(Manα1-2Manα1-2Manα1-3)Manβ1-4GlcNAcβ1-4GlcNAc-PA
M7D: Manα1-6(Manα1-2Manα1-3)Manα1-6(Manα1-2Manα1-3)Manβ1-4GlcNAcβ1-4GlcNAc-PA
M8A: Manα1-2Manα1-6(Manα1-3)Manα1-6(Manα1-2Manα1-2Manα1-3)Manβ1-4GlcNAcβ1-4GlcNAc-PA
M8C: Manα1-6(Manα1-2Manα1-3)Manα1-6(Manα1-2Manα1-2Manα1-3)Manβ1-4GlcNAcβ1-4GlcNAc-PA
M9A: Manα1-2Manα1-6(Manα1-2Manα1-3)Manα1-6(Manα1-2Manα1-2Manα1-3)Manβ1-4GlcNAcβ1-4GlcNAc-PA
PA-: pyridylamino
PBS: phosphate βuffer saline
PNGase: peptide: N-glycanase
RP-HPLC: reverse-phase HPLC
TFA: trifluoroacetic acid
Xyl: D-xylose
